# An esophageal submucosal tumor treated with submucosal tunneling endoscopic resection: an unexpected result

**DOI:** 10.1093/gastro/goaa073

**Published:** 2020-12-25

**Authors:** Ming-Yan Cai, Mariana Ferreira Cardoso, Yan Zhu, Yun-Shi Zhong, Ping-Hong Zhou

**Affiliations:** 1 Endoscopy Center, Zhongshan Hospital, Fudan University, Shanghai, P.R. China; 2 Gastroenterology Department, Hospital Professor Doutor Fernando Fonseca, Amadora, Portugal

## Case presentation

**Figure 1.**
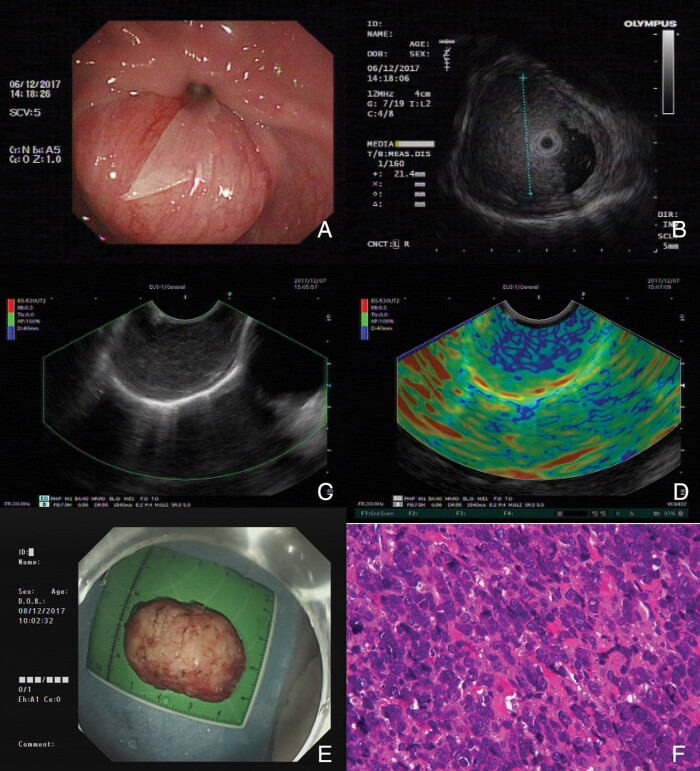
Preoperative evaluation and post-operative pathology of an esophageal submucosal tumor (SMT). (**A**) A SMT in the lower esophagus is identified on esophagogastroduodenoscopy. (**B**) Mini-probe endoscopic ultrasonography (EUS) shows a hypoechoic lesion mainly located in the submucosal layer and that originating from the superficial muscularis propria layer. (**C**) Linear EUS with Doppler shows little flow in the tumor. (**D**) The elastography shows a predominantly blue pattern suggesting high tissue stiffness. (**E**) The tumor was resected *en bloc* by submucosal tunneling endoscopic resection (STER). (**F**) Post-operative pathology reveals a poorly differentiated neuroendocrine carcinoma (small-cell carcinoma) (Hematoxylin-Eosin staining, x 200).

A 66-year-old female patient underwent esophagogastroduodenoscopy (EGD) in our center because of occasional dysphagia, and a large submucosal tumor (SMT) was identified 32–36 cm from the incisors, occupying nearly half of the luminal circumference **(Figure 1A)**. Mini-probe endoscopic ultrasonography (EUS) revealed a hypoechoic submucosal lesion, measuring 20 × 15 mm and originating from the superficial muscularis propria layer **(Figure 1B).** It was covered by normal-appearing mucosa. The patient underwent EUS (SU-9000, Fujifilm, Japan) for further investigation **(Figure 1C).** EUS elastography showed high tissue stiffness with a predominantly blue pattern **(Figure 1D)** and there was no enhancement after injection of intravenous contrast. Overall, these findings were consistent with those of a submucosal tumor (SMT) of the lower esophagus originating in the muscularis propria, possibly a leiomyoma.

The patient underwent *en bloc* resection of the tumor by submucosal tunneling endoscopic resection (STER). The first step of the procedure consisted of a submucosal injection 5 cm proximally to the lesion. A longitudinal mucosal incision and subsequent submucosal tunneling were performed using the Hybrid Knife (ERBE, Germany). Endoscopic *en bloc* resection was then carried out using both the Hybrid Knife and the IT Knife (Olympus, Japan). After retrieval of the endoscopic specimen measuring 40 x 20 mm **(Figure 1E)** and adequate hemostasis of the tunnel, the incision site was closed using hemostatic clips.

However, histology revealed a poorly differentiated neuroendocrine carcinoma (small-cell carcinoma) invading the submucosal layer with lymphovascular invasion **(Figure 1F)**. Immunohistochemistry staining was positive for CD56, synaptophysin, CgA and CK-PAN and negative for SSTR2, SSTR5, and p53. The Ki-67 score exceeded 60%. The patient was suggested to receive further surgical treatment. However, she did not seek any treatment. On a 3-month follow-up, investigation revealed distant metastases to both liver lobes and the left chest wall so the patient was referred for palliative care.

## Discussion

Esophageal neuroendocrine neoplasms (NENs) are extremely rare tumors, accounting for only 1.0%–1.4% of all gastroenteropancreatic NENs [1, 2]. The WHO 2010 Classification, incorporating both the mitotic rate and the Ki-67 index, divides NENs into low-grade (G1) neuroendocrine tumor (NET), intermediate-grade (G2) NET, and high-grade (G3) neuroendocrine carcinoma (NEC). NECs are further classified into small-cell NEC, accounting for 88%–94% of cases [3, 4], and large-cell NEC. Different from other NENs showing an indolent growth pattern, esophageal NECs have an extremely aggressive biology and hence prognosis is very poor. In fact, esophageal NECs share many similarities with small-cell carcinoma of the lung [5], which reflects on the treatment modalities for the esophageal counterpart. No definite treatment recommendations can be made due to the paucity of data; it generally comprises surgery for limited disease, possibly combined with chemo and radiotherapy, and palliative chemotherapy for extensive disease [6].

The main endoscopic finding for esophageal NEC is a protruding or localized lesion with or without ulceration in the center [7]. To the best of our knowledge, this is an unusual case presenting as an esophageal SMT originating from the muscularis propria layer while preserving the mucosal surface, mimicking lesions such as esophageal leiomyoma, the most commonly found esophageal SMT, or rarer tumors including gastrointestinal stromal tumor (GIST) and schwannoma. In fact, in preoperative ultrasonographic evaluation, this lesion appeared as a hypoechoic tumor originating from the fourth layer, with high stiffness and no contrast enhancement. Contrast-enhanced EUS (CE-EUS) recently emerged as a strategy to differentiate gastrointestinal SMTs, after it was implemented for the characterization of pancreatic tumors [8]. Typically, leiomyomas present as hypo-enhancing lesions, whereas GISTs are hyper-enhancing. Similar to GISTs, NENs are also hyper-enhancing owing to their rich vascularization. However, there is little literature describing the CE-EUS pattern for esophageal NEC. In our patient, the aforementioned pattern of no enhancement seems to be unexpected. Esophageal NECs generally present either an exophytic polypoid or ulcerated gross appearance, often with surface necrosis [4]. Although foregut NENs are known to grow into the submucosa [9], such a deep growth pattern with preserved mucosal surface as found in our case has not been previously reported.

Several cases of endoscopically removed small low-grade esophageal NENs have been described, usually through endoscopic mucosal resection (EMR) or endoscopic submucosal dissection (ESD) [6]. Since it was first described in 2012 [10], the STER technique became standard for the resection of esophageal SMTs in some endoscopic centers. Although in the present case endoscopic *en bloc* resection was technically feasible through STER, we emphasize that this cannot be routinely recommended in such advanced malignancies. It also arouses a dilemma for those technically resectable SMTs—a pre-procedure EUS-guided fine-needle aspiration (EUS-FNA) for diagnosis is a must or not?

In conclusion, our case illustrates that, despite recent advances in the preoperative assessment of gastrointestinal SMTs, a definite diagnosis can only be obtained through histopathologic examination of the resected specimen. In fact, even though, in this case, evidence pointed to a benign lesion such as a leiomyoma, diagnosis turned out to be that of a malignant lesion with poor prognosis. We also describe previously unrecognized features of esophageal NEC—a rare and aggressive disease that remains incompletely understood.
